# Molecular Breeding for Nutritionally Enriched Maize: Status and Prospects

**DOI:** 10.3389/fgene.2019.01392

**Published:** 2020-02-21

**Authors:** Boddupalli M. Prasanna, Natalia Palacios-Rojas, Firoz Hossain, Vignesh Muthusamy, Abebe Menkir, Thanda Dhliwayo, Thokozile Ndhlela, Felix San Vicente, Sudha K. Nair, Bindiganavile S. Vivek, Xuecai Zhang, Mike Olsen, Xingming Fan

**Affiliations:** ^1^ International Maize and Wheat Improvement Center (CIMMYT), Nairobi, Kenya; ^2^ CIMMYT, Texcoco, Mexico; ^3^ ICAR-Indian Agricultural Research Institute (IARI), New Delhi, India; ^4^ International Institute of Tropical Agriculture (IITA), Ibadan, Nigeria; ^5^ CIMMYT, Harare, Zimbabwe; ^6^ CIMMYT, ICRISAT, Hyderabad, India; ^7^ Institute of Crop Sciences, Yunnan Academy of Agricultural Sciences (YAAS), Kunming, China

**Keywords:** biofortification, quality protein maize, provitamin A, kernel zinc, vitamin E

## Abstract

Maize is a major source of food security and economic development in sub-Saharan Africa (SSA), Latin America, and the Caribbean, and is among the top three cereal crops in Asia. Yet, maize is deficient in certain essential amino acids, vitamins, and minerals. Biofortified maize cultivars enriched with essential minerals and vitamins could be particularly impactful in rural areas with limited access to diversified diet, dietary supplements, and fortified foods. Significant progress has been made in developing, testing, and deploying maize cultivars biofortified with quality protein maize (QPM), provitamin A, and kernel zinc. In this review, we outline the status and prospects of developing nutritionally enriched maize by successfully harnessing conventional and molecular marker-assisted breeding, highlighting the need for intensification of efforts to create greater impacts on malnutrition in maize-consuming populations, especially in the low- and middle-income countries. Molecular marker-assisted selection methods are particularly useful for improving nutritional traits since conventional breeding methods are relatively constrained by the cost and throughput of nutritional trait phenotyping.

## Introduction

**Figure 1:**
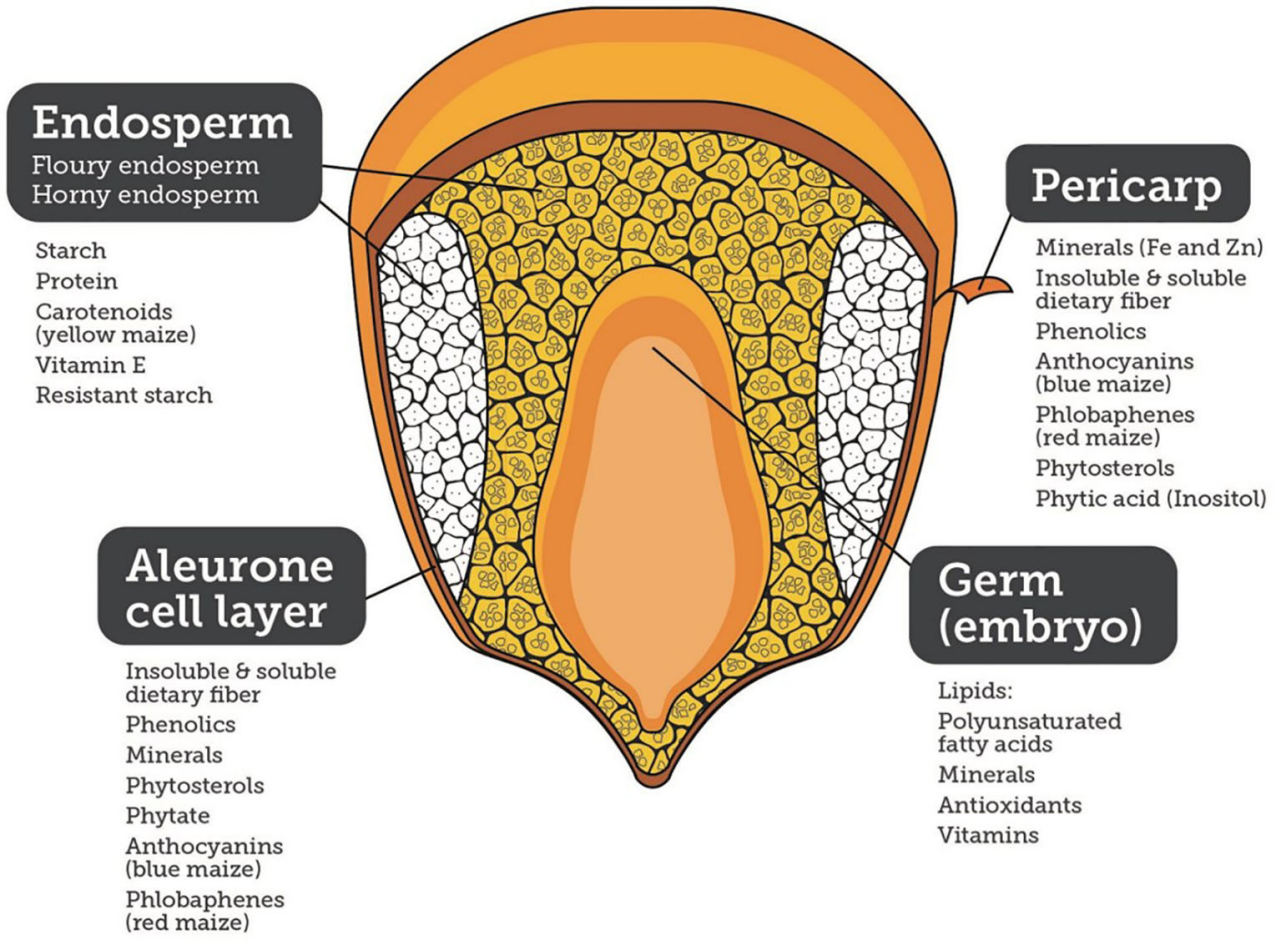
Nutritional quality of different components of a maize kernel.

Maize and its products constituted 30% of the food supply in the Americas, 38% in Africa, and 6.5% in Asia, and thus, is a major source of food security and economic development. Maize is major staple food and the most important energy source in sub-Saharan Africa (SSA) with intakes ranging from 50 to >330 g/person/day, and providing daily energy, protein, and micronutrients. In Latin America, maize consumption ranges from 50 to >300 g/person/day. Additionally, maize is part of the livestock-to-meat cycle across the world (Tanumihardjo et al., 2019). In addition to calories, maize is a source of micronutrients and phytochemicals, such as phenolics, carotenoids (yellow and orange maize), anthocyanins (blue, purple, and black maize), phlobaphenes (red maize), insoluble and soluble dietary fiber, and polar and non-polar lipids, providing health benefits and helping prevent diseases (**Figure 1**).

High per capita consumption of maize and limited diet diversification in several countries across Africa, Latin America, and Asia signifies that the greater part of people’s diets in these countries lack some of the essential micronutrients, such as zinc (Zn), vitamin A, and vitamin E, as well as essential amino acids, such as lysine and tryptophan. More than two billion people in Asia, Africa, and Latin America suffer from one or more micronutrient deficiencies that commonly lead to retarded physical growth, impaired cognitive development, complications during pregnancy, diminished work and income earning capacity, and increased risk of morbidity and mortality (Bailey et al., 2015; Rautiainen et al., 2016).

The concentrations of various nutrients in maize kernels depend on the genetic background or the genotype, agronomic management, interaction between genotype and the environment, and post-harvest handling (Ekpa et al., 2019). Although the concentrations of most micronutrients in commonly used maize worldwide are not enough to have a nutritional impact on consumers, there is large genetic variation in maize that allows development of improved cultivars with higher concentrations of certain micronutrients, through biofortification (Bouis and Saltzman, 2017). Additional or complementary technologies in crop management and food science can also contribute to enhancing the nutritional impact of maize-based diets (Nuss and Tanumihardjo, 2011; Ekpa et al., 2018).

Maize scientists have been developing improved cultivars with enhanced nutritional value, such as quality protein maize (QPM) rich in two essential amino acids (Prasanna et al., 2001; Atlin et al., 2011); orange maize biofortified with provitamin A carotenoids (Pixley et al., 2013); and high-Zn-enhanced maize (Andersson et al., 2017). Higher content of lysine and tryptophan, kernel Zn, and provitamin A have been successfully increased in maize through conventional breeding. In SSA, the spread of QPM cultivars has been faster than in Asia, mainly because maize is predominantly used as food in SSA. In Asian countries including India, maize grains are utilized more as poultry feed (~60–70% of the produce), and synthetic lysine and tryptophan are added as supplements. Besides grain corn, nutritional enrichment of sweet corn is another possibility. Sweet corn has emerged as an important source of income for farmers in Asia; US$1,034 million worth of preserved sweet corn is imported globally, while the same for frozen sweet corn was $423 million (FAOSTAT, 2017).

There are now significant opportunities for more effectively developing nutritionally enriched cultivars of both grain and specialty corn, due to various factors, including availability of large genetic diversity for the target traits, advances in understanding key biochemical pathways for metabolite biosynthesis, analytical tools for screening germplasm for quality traits, and the possibilities to utilize molecular markers and genome editing approaches to accelerate product development (Reynolds et al., 2019). In this review, we have highlighted the recent advances in breeding for nutritionally enriched maize, especially in the tropics.

## Maize With Enhanced Protein Quality

### Extent of Quality Protein Deficiency

Essential amino acids such as lysine and tryptophan not only act as building blocks of proteins but also serve as neuro-transmitters. The recommended daily allowance of lysine is 30 mg kg^−1^ body weight for adults, and 35 mg kg^−1^ body weight for children. As regards tryptophan, the daily requirements are 4 and 4.8 mg kg^−1^ body weight per day for adults and children, respectively (WHO/FAO/UNU, 2007). Deficiency of these amino acids leads to reduced appetite, delayed growth, impaired skeletal development, and aberrant behavior (Tome and Bos, 2007).

Several smallholders and their families in the low- and middle-income countries in SSA and Latin America are dependent on maize not only for their calorie requirement but also for dietary protein. Such populations, along with their monogastric livestock, run the risk of incurring health problems associated with amino acid deficiency, as maize is deficient in two essential amino acids, lysine and tryptophan. Improving the quality of maize endosperm protein by increasing its lysine and tryptophan content has, therefore, been a goal of maize breeding programs of the International Maize and Wheat Improvement Center (CIMMYT), the International Institute of Tropical Agriculture (IITA), and several national programs. This has led to development of an array of QPM cultivars having approximately twice the content of tryptophan (0.07–0.08% in flour) and lysine (0.25–0.40% in flour) compared to conventional maize cultivars (tryptophan: 0.03–0.04% in flour; lysine: 0.15–0.20% in flour), and consequently having greatly improved nutritional quality (Bressani, 1992; Bjarnason and Vasal, 1992; Zarkadas et al., 1995; Prasanna et al., 2001).

### Quality Protein Maize Genetics and Breeding

Development of QPM cultivars involves manipulating three distinct genetic systems (Bjarnason and Vasal, 1992; Prasanna et al., 2001): 1) the simple recessive allele of *opaque2* (*o2*) gene in homozygous condition; 2) modifiers/enhancers of the *o2o2*-containing endosperm to confer higher lysine and tryptophan; and 3) genes that modify the *opaque2-*induced soft endosperm to hard endosperm. Phenotypic selection followed by biochemical analysis are required to select desirable genotypes that combine the three systems. Genomic screening methods may be used to enhance the efficiency of this selection process (Babu et al., 2005).

The deployment of *o2* located on chromosome 7 along with the endosperm modifiers led to the successful commercialization of diverse QPM hybrids worldwide with enhancement of both lysine (from 1.6–2.6 to 2.7–4.5% in protein) and tryptophan (from 0.2–0.6 to 0.5–1.1% in protein) (Vivek et al., 2008). The search for a novel mutation that can be successfully utilized to develop high lysine maize continued in the new millennium until Yang et al. (2005) reported another recessive mutant from Robertson’s Mutator stocks and named it as *opaque16* (*o16*). Simple sequence repeats (SSRs), *umc1141* and *umc1149* were identified as the closely linked markers to *o16* (located on chromosome 8) using F_2_ mapping population developed between Chinese inbreds, QCL3024 (*o16*) and QCL3010 (wild type). *o2o2/o16o16* was reported to increase lysine by 30% over *o2o2* or *o16o16* alone (Yang et al., 2005). Sarika et al. (2017) studied two F_2_ populations derived by crossing wild type (CML533 and CML537) and *o16*-donor line (QCL3024). Genotypes with *o16o16* possessed on average nearly two-fold more lysine (0.247% in flour) and tryptophan (0.072% in flour) compared to normal maize (lysine 0.125% and tryptophan 0.035%, in flour), although the wide variation for the two traits across populations (lysine: 0.111–0.376% in flour; tryptophan: 0.027–0.117% in flour) suggested the possible influence of modifier loci.

Yang et al. (2013) reported that average lysine in *o16o16*–based BC_2_F_4_ seeds was 0.352%; some of the segregants (*o16o16*) possessed comparable lysine and tryptophan usually observed in *o2o2* genotypes, thereby suggesting that *o16* can be used as replacement to *o2* in the QPM breeding program. Sarika et al. (2018a) reported that the seed endosperm of *o16o16* was vitreous and phenotypically similar to wild type (*O16O16*). The mutant did not influence the degree of kernel opaqueness in *o2o2* genetic background as opaqueness in *o2o2/O16O16* and *o2o2/o16o16* was similar. Grain hardness of *o16o16* was comparable with the normal and QPM maize. The pattern of microscopic organization of proteinaceous matrix and starch granules, and zein profiling of the storage protein in *o16o16* were found to be similar with normal maize endosperm, but distinct from the *o2o2*-soft genotype.

### Molecular Breeding for Developing Improved Quality Protein Maize Cultivars

A strong recognition of the relevance of QPM came through the award of World Food Prize to Surinder Vasal and Evangelina Villegas in the year 2000, leading to a resurgence in QPM breeding and release of cultivars [both open-pollinated varieties (OPVs) and hybrids] across SSA, Asia, and Latin America. In Asia, more than 40 QPM cultivars have been developed and released through conventional breeding, with India, China, Indonesia, and Vietnam having the highest number of releases. Several of the QPM cultivars were initially identified through CIMMYT International Trials distributed to partners, while national programs in India and China further released an array of QPM cultivars with introgression of the QPM trait in commercially relevant genetic backgrounds.

In India, marker-assisted backcross breeding (MABB) for *o2* led to the development and release of a single-cross QPM hybrid, ‘Vivek QPM-9’ in 2008 (Gupta et al., 2013). It possessed 41% more tryptophan and 30% more lysine over the original hybrid (Vivek Hybrid-9). Later, *o2* allele was introgressed into the parental inbreds of three popular non-QPM hybrids. These hybrids *viz.*, ‘Pusa HM-4 Improved’, ‘Pusa HM-8 Improved’, and ‘Pusa HM-9 Improved’ have been released in 2017 for commercial cultivation in India (Hossain et al., 2018a), possessing nearly double the concentrations of lysine and tryptophan as compared to normal maize. These four hybrids are in the flint background, and did not show any yield penalty over the original hybrids (Yadava et al., 2018). At CIMMYT several inbreds were converted to QPM versions (e.g., CML244Q, CMl246Q, CML349Q, and CML354Q) using MABB; the grain yield performance of these QPM versions were at par with the original versions. Several institutions under the Indian Council of Agricultural Research (ICAR) and State Agricultural Universities (SAUs) are now using marker-assisted selection (MAS)-based breeding methods for developing new QPM cultivars (Hossain et al., 2019).

The Institute of Crop Science, Chinese Academy of Agricultural Sciences (CAAS) developed diverse QPM inbred lines by marker-assisted backcrossing in different genetic backgrounds (Tian et al., 2004; Jiang et al., 2005). Zhang et al. (2010) combined *o2* and *o16*, and reported an enhancement of 23% lysine in *o2o2/o16o16* progeny over the *o2o2* inbred comparison. Zhang et al. (2013) further pyramided *o2* and *o16* in waxy genetic background, and reported higher accumulation of lysine (0.616% in flour) in the pyramided lines compared to *o2o2* segregants (0.555% in flour). Yang et al. (2013) introgressed the *o16* allele from QCL3024 into two Chinese waxy lines, QCL5019 and QCL5008 using MAS. The *o16o16*-based waxy inbreds possessed 16–27 and 18–28% higher lysine than the waxy parents, respectively. Liu et al. (2016) developed the functional marker qγ27 linked with endosperm modification, which provides an important technical support in breeding QPM, although laboratory analysis is still needed for quantification of lysine and tryptophan.

Marker-assisted breeding has also been initiated in India to combine both *o2* and *o16* for developing QPM hybrids with enhanced levels of lysine and tryptophan (Sarika et al., 2018b; Chand et al., 2019). Sarika et al. (2018b) introgressed *o16* into the parental lines of four commercial QPM hybrids (HQPM-1, HQPM-4, HQPM-5, and HQPM-7) released in India, using MABB. Reconstituted hybrids showed an average enhancement of 49 and 60% in lysine and tryptophan over the original hybrids, with highest enhancement amounting 64 and 86%, respectively. The degree of endosperm modification in these hybrids was similar to the original QPM hybrids. The grain yield potential of these *o2o2/o16o16* hybrids was also at par with the original *o2o2*-based released hybrids, indicating that *o16* could be a novel genetic resource for enhancing lysine and tryptophan without influencing the degree of kernel opaqueness and grain yield potential.

## Provitamin A-Enriched Maize

### Extent of Vitamin a Deficiency

Vitamin A plays a vital role in vision, and lack of it causes night blindness and partial or even complete loss of eyesight in humans. An adult non-pregnant and non-lactating woman requires 500 μg day^−1^ of vitamin A, while children of 4–6 years require 275 μg day^−1^ (Andersson et al., 2017). SSA has recorded the highest rates of vitamin A deficiency or VAD (48%), followed by South Asia (42%) (UNICEF, 2016). Pregnant women, breast-feeding mothers, and their children younger than 5 years of age are at the highest risk of having VAD (Bailey et al., 2015). In some countries in SSA, VAD in the most vulnerable populations is associated with reduced immune response, which can lead to increased infections, such as diarrhea, measles, or respiratory infections, which may either decrease provitamin A intake through reduced appetite or deplete existing vitamin A stores through excessive metabolism (Alvarez et al., 1995; Mitra et al., 1998).

### Breeding Provitamin A-Enriched Maize

Maize has been targeted as one of the major food crops for provitamin A (PVA) enrichment and delivery under the HarvestPlus Program (Pfeiffer and McClafferty, 2007; Bouis and Welch, 2010). The main objective of the PVA enrichment in maize breeding program has been to develop high-yielding, provitamin A-enriched maize cultivars that are profitable to farmers and acceptable to the consumers, and with proven effectiveness in reducing vitamin A deficiency (Bouis and Welch, 2010). Yellow maize naturally accumulates PVA carotenoids, including α-carotene, β-carotene (BC), and β-cryptoxanthin (BCX), which can be metabolically converted to active vitamin A in the human body (Asson-Batres and Rochette-Egly, 2016). However, kernels of yellow maize cultivars commonly grown by farmers contain less than 2 μg g^−1^ of PVA (Ortiz-Monasterio et al., 2007; Pixley et al., 2013), which is insufficient to meet the recommended daily requirement in a diet (Institute of Medicine, 2012). Considerable efforts have thus been made to increase the concentrations of PVA carotenoids in maize through conventional and molecular marker-assisted breeding (Pixley et al., 2013; Giuliano, 2017; Andersson et al., 2017; Menkir et al., 2017).

**Figure 2:**
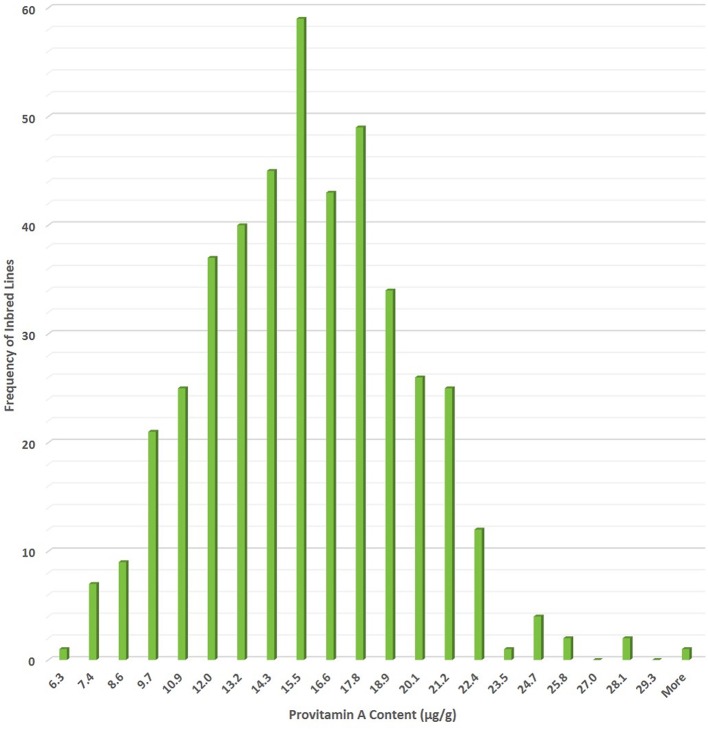
Extent of genetic variation for provitamin A content (µg/g) in International Maize and Wheat Improvement Center (CIMMYT) maize inbred lines developed under HarvestPlus-maize biofortification program.

The breeding target for PVA in maize deemed sufficient to impact human health was set at 15 µg g^−1^ of BC equivalents, by a multidisciplinary team that included plant breeders, plant geneticists, biochemists, nutritionists, and food processing specialists (Hotz and McClafferty, 2007). PVA content was estimated as one part of BC plus one and a half part of BCX, based on the number of unmodified β-rings and number of retinol molecules that can be derived from them (Von Lintig, 2010; Wurtzel et al., 2012). The first step in developing PVA maize was screening of more than 1,500 genotypes for their carotenoid profiles. The majority of inbred lines surveyed had BCX and BC content averaging from 1 to 2 µg g^−1^ and PVA content of 2–3 µg g^−1^ (Ortiz-Monasterio et al., 2007; Menkir et al., 2008), but a few temperate lines had PVA levels approaching 15 µg g^−1^ (Ortiz-Monasterio et al., 2007). Nonetheless, the levels of PVA carotenoids in adapted tropical and sub-tropical maize inbred lines were far below the breeding target of 15 µg g^−1^ of PVA set for maize (Bouis et al., 2011), emphasizing the need for accessing and mining novel sources of favorable alleles to boost PVA concentration to new levels. The less complex nature of control of provitamin A content, high heritability, mode of inheritance regulated primarily by additive genetic effects, and statistically non-significant correlation between PVA and agronomic performance suggested that concurrent improvements of PVA carotenoids and grain yield would be possible (Suwarno et al., 2014; Menkir et al., 2018; Ortiz-Covarrubias et al., 2019). Through systematic breeding efforts, significant improvement has been made in enhancing provitamin A content of tropical maize inbred lines developed at CIMMYT (**Figure 2**).

Selection for PVA content has focused mostly on increasing BC; however, new evidence suggests that BCX may be more bioavailable (Howe and Tanumihardjo, 2006; Schmaelzle et al., 2014; Sugiura et al., 2014) and less susceptible to degradation than BC. Recent studies (Dhliwayo et al., 2014; Ortiz et al., 2016; Taleon et al., 2017; Sowa et al., 2017) suggested that breeding for PVA carotenoids should aim to increase BCX more than BC due to the increasing evidence of low stability of BC, higher BCX bioavailability compared to BC, and BCX’s similar bioconversion and bioefficacy to BC (Schmaelzle et al., 2014), in addition to the genetic diversity found for BCX (Suwarno et al., 2015; Menkir et al., 2018). Breeding efforts have started in this regard and inbred lines have been developed and are currently being used in the development of new hybrids and synthetics. Research focused on minimizing carotenoid degradation is also needed.

### Molecular Breeding for Provitamin A Enrichment

The carotenoid biosynthesis pathway is well-studied and genes controlling key steps in the pathway have been cloned and characterized. Allelic variation in key genes has been exploited to identify and develop DNA markers associated with BC and BCX, the main carotenoids with PVA activity. Use of MAS in combination with high-performance liquid chromatography (HPLC)/ultra-performance liquid chromatography (UPLC) analysis has been more effective than biochemical screening alone (Zhang et al., 2012; Simpungwe et al., 2017).

Most of the breeding work until about 2011 was based on phenotypic selection and biochemical analysis was used to quantify PVA levels in selected lines (Pixley et al., 2013). Polymorphisms were identified in two genes: *β-carotene hydroxylase1* (*CrtRB1*) which catalyzes the hydroxylation of BC to BCX (Yan et al., 2010), and *lycopene epsilon cyclase* (*LcyE*), which converts lycopene to zeta (ζ)-carotene and ultimately to α-carotene (Harjes et al., 2008). The favorable allele at *CrtRB1* reduces hydroxylation of BC into BCX whereas *LcyE* reduces flux into the α-branch of the pathway (Von Lintig, 2010). Both alleles increase PVA content, but the favorable *CrtRB1* allele is more effective in increasing PVA content than the favorable *Lcyє* allele (Yan et al., 2010; Babu et al., 2013).

Three functional polymorphisms were thus identified in the two genes: CrtRB1-5′TE, CrtRB1-3′TE, and LcyE 3′Indel. Babu et al. (2013) evaluated these three polymorphisms in CIMMYT maize germplasm and reported more than two-fold increase in BC associated with the favorable allele at CrtRB1-3′TE, irrespective of the genotype at CrtRB1-5′TE. The favorable allele LcyE 3′Indel reduced the ratio of α to β-branch carotenoids by up to 30%, without a notable increase in BC or PVA content. Effectiveness of the CrtRB1-3′TE polymorphism in increasing PVA content was also demonstrated by Zunjare et al. (2018). The maximum PVA content that can be achieved by selecting for both the CrtRB1-3’TE and LcyE 3′Indel polymorphisms is unknown, partly because of a large genetic background effect or epistasis (Babu et al., 2013). Nevertheless, donor germplasm with >20 µg g^−1^ have been developed using MAS targeting the favorable alleles for the two polymorphisms in a pedigree breeding scheme. Foreground MAS selection was done in segregating F_2_ or F_3_ populations, selecting seeds that had the homozygous favorable alleles at the CrtRB1-3′TE and LcyE-3′Indel polymorphisms. However, combining foreground and background MAS may be preferable to reduce linkage drag considering that most of the PVA maize donor germplasm is agronomically inferior (Zunjare et al., 2018; Ortiz-Covarrubias et al., 2019).

During 2011 to 2015, CIMMYT’s PVA breeding program at Mexico was manually chipping and genotyping 10,000 seeds per season and selecting homozygous individuals (~20–25%) for planting. This process saved land and labor resources by ensuring that only individual plants that carried the favorable alleles were planted while enabling development of inbred lines that reached or exceeded the breeding target coupled with good yield potential, disease resistance, grain quality traits, and other agronomic traits. However, the gel-based assays were expensive and the manual seed chipping method was laborious and time-intensive. Scientists at CIMMYT and IITA developed the single nucleotide polymorphism (SNP) markers, which have been used to develop an automated high-throughput assay with an external service provider. Currently, in SSA, MAS for the favorable CrtRB1-3′TE is done in combination with MAS for *maize streak virus 1* (MSV1) that is associated with resistance to the viral disease maize streak virus (Nair et al., 2015).

## High-Zinc Maize

### Extent of Zinc Deficiency

According to the World Health Organization, Zn deficiencies affected 17% of the global population (www.harvestplus.org). Zn deficiency is widespread and particularly prevalent in Africa, the eastern Mediterranean, and South and Southeast Asia (Caufield et al., 2004). In young children, it increases the risk of diarrheal disease, pneumonia, malaria, and mortality from those diseases. Based on the estimated average requirement (EAR) of 1,860 µg day^−1^ of Zn in maize, the breeding target established by HarvestPlus for maize was 33 µg g^−1^ (Bouis and Welch, 2010). Andersson et al. (2017) indicated a new EAR of 2,960 µg day^−1^ for women and 1,390 µg day^−1^ for children. The baseline content for Zn in maize is about 20 µg g^−1^; assuming 90% retention of Zn after processing and 25% bioavailability, at least 33 ppm of Zn needs to be accumulated in the maize grains. Thus, an increase of at least 13 µg g^−1^ is targeted by breeding, which is achievable due to significant genetic variation for kernel-Zn concentration in tropical maize germplasm. With the availability of high-Zn tropical maize genotypes, studies on nutrient retention, bioavailability, and efficacy are being planned.

### Breeding High-Zn Maize Cultivars

Biofortification of maize kernels with high-Zn has been undertaken at CIMMYT and IITA, in active partnership with public and private sector partners. Substantial genetic variation for kernel-Zn (4–96 ppm) was found in tropical maize germplasm (Bänziger and Long, 2000; Ortiz-Monasterio et al., 2007; Menkir, 2008; Chakraborti et al., 2011; Prasanna et al., 2011; Hindu et al., 2018), including landraces, inbreds, hybrids, and OPVs. Unlike crops like wheat and rice (Guzmán et al., 2014), in maize no statistically significant correlation was observed between kernel Zn and Fe contents. In fact, high Zn maize normally contain between 18 and 20 ppm Fe, which is the average content in maize kernel. In addition, factors like limited genetic variation and the high target levels needed to reach nutritional impact in the consumers affected Fe biofortification in maize using breeding strategies.

Breeding efforts at CIMMYT and IITA were initiated to meet the target level of 33 ppm kernel-Zn (dry weight) (Bouis et al., 2011). The initial focus has been on Latin American countries including Guatemala, Nicaragua, Honduras, and Colombia, and Western African countries, including Ghana, Benin, and Nigeria. Zn biofortification breeding at CIMMYT has utilized white-endosperm and high kernel-Zn lines with QPM background. Three QPM CIMMYT Maize Lines (CMLs)—CML176, CML491, and CML492—were found to be particularly important for improving kernel Zn in tropical maize, and have been used extensively as founder lines in pedigree-based selection. These elite QPM lines were derived from CIMMYT maize Population 62 (white flint QPM) and Population 63 (white dent QPM), which underwent several cycles of intra-population recurrent selection in the 1980s (CIMMYT, 1998). Population 62 traces back to ETO composite, whose main components were the tropical Colombian landraces, Comun and Chococeño, and the Venezuelan landraces, Puya and Cubano Amarillo. Population 63 traces back to Tuxpeño-1 composite, whose main component was the Mexican landrace Tuxpeño (CIMMYT, 1998). Adapted yellow and white endosperm maize inbred lines derived from broad-based populations, bi-parental crosses, and backcrosses with high Zn content have been used for generating pedigree populations at CIMMYT and IITA to develop new high Zn inbred lines.

Interestingly, above-average concentration of Zn in the kernels was found in the QPM germplasm as compared to non-QPM/normal maize germplasm (Chakraborti et al., 2009; Chakraborti et al., 2011). However, not all QPM germplasm is high in kernel Zn, and it is possible to have some non-QPM germplasm with high kernel Zn. Zn plays an important role in tryptophan biosynthesis, which is increased in QPM. Using 923 lines to conduct genome-wide association studies (GWAS) for kernel Zn, Hindu et al. (2018) reported that only 31 were QPM or had QPM background and 33.3% had Zn values higher than 30 µg g^−1^on dry weight (DW) basis. In contrast, out of the 892 non-QPM used in the panel, 19.9% had values higher than 30 µg g^−1^DW, and about 6% of them had values higher than the breeding target (33 µg g^−1^DW). Taken together, these results indicate great potential to develop high Zn maize alone or in combination with better protein quality in biofortification programs.

### Molecular Breeding for Kernel-Zn Enrichment

Quantitative trail locus (QTL) mapping studies confirmed that kernel Zn accumulation is under the control of several genetic loci (Qin et al., 2012; Baxter et al., 2013). Complexity of the trait is further increased due to higher environment and genotype × environment interaction (GEI) effects. Over the past few years, genomic regions influencing Zn concentration have been detected through GWAS and biparental QTL mapping in maize. GWAS of 923 tropical/sub-tropical CIMMYT maize inbred lines, phenotyped at three locations in Mexico and genotyped using high density genotyping by sequencing (GBS), identified a total of 20 SNPs significantly associated with kernel-Zn. This effort constitutes the first large-scale screening of tropical/sub-tropical public germplasm for kernel Zn. A set of 11 SNPs identified in GWAS have been subsequently validated in independent biparental populations using single factor QTL analysis, and some of these SNPs explained a relatively high proportion of variance (Hindu et al., 2018).

Qin et al. (2012) identified three stable QTLs for kernel-Zn concentrations in two populations across two environments. Šimić et al. (2012) identified two QTLs on chromosomal bin 3.05 and 4.08 which explained small percentage of the variation in a temperate biparental population phenotyped at two locations. Jin et al. (2013) conducted a meta-QTL study with QTL mapping studies published for kernel Zn and many related minerals, and identified nine meta-QTLs across the maize chromosomes that could have an influence in kernel-Zn concentration. Earlier studies identified some major QTLs for kernel Zn trait in chromosomal bins 3.04 (Qin et al., 2012), 4.06, 5.04 (Jin et al., 2013), and 9.06-07 (Qin et al., 2012; Jin et al., 2013); significant SNP markers were identified through CIMMYT GWAS and validation studies. To assess their utility in breeding program, these SNPs were further analyzed in parental lines selected for high kernel-Zn at CIMMYT, including analysis of the frequency of favorable alleles in the breeding pool. The favorable allele frequency ranged between 0.09 and 0.94 in CIMMYT’s breeding lines for these SNPs/haplotypes. Three haplotypes were selected on chromosomes 5, 7, and 9 based on their favorable allele frequency and effect size of favorable alleles. In a set of 1,880 breeding lines from pedigree crosses entering stage 1 testing in the kernel-Zn breeding pipeline, it was seen that the selection for the three favorable haplotypes increased the population mean kernel-Zn content by 16.3% (Sudha K Nair, unpublished).

Genomic selection (GS) has been demonstrated as an effective approach to accelerate genetic gain in maize breeding for improvement of complex traits (Zhang et al., 2015; Cao et al., 2017; Yuan et al., 2019). The genomic prediction accuracy for kernel Zn content in maize has been estimated at CIMMYT in different types of maize populations with repeat amplification sequencing and GBS markers. Moderate to high prediction accuracies, ranging from 0.35 to 0.65, were observed across different types of populations and genotyping platforms (Xuecai Zhang, unpublished). Thus, kernel Zn content in maize could be improved by implementing MAS and GS in a stepwise fashion, where the SNPs/haplotypes detected and validated in the association mapping and linkage mapping analyses can be used in forward breeding at an early generation when there are larger numbers of selection candidates, followed by genomic selection at advanced stages of breeding or the SNPs/haplotypes can be fitted as fixed effects in GS models to improve prediction accuracy.

### Low Phytate Maize Genotypes for Enhancing Kernel-Zn Bioavailability

Breeding for high kernel Zn has been a challenge to the maize breeders worldwide, primarily due to involvement of large number of loci with minor effects and existence of very high GEI (Colangelo and Guerinot, 2006). Moreover, bioavailability of Zn in maize grains is only 20% in the human gut (Andersson et al., 2017). The major impediment of low bioavailability of Zn has been the presence of phytic acid/phytate that constitutes nearly 75–80% of the total phosphorus in maize grains (Raboy, 2001). Maize kernels generally contains ~3.2 mg day^−1^ of phytic acid with a range of 2.4 to 4.1 mg day^−1^ (Lorenz et al., 2007). Phytate being negatively charged has a strong tendency to chelate positively charged metal ions, such as Zn, thereby resulting in highly insoluble salts with poor bioavailability of the nutrient (Zhou and Erdman, 1995). Monogastric animals including humans, poultry, and swine cannot digest phytic acid in their gut, since they lack phytic acid hydrolyzing enzyme phytase. Phytate is thus expelled directly to the environment through excreta posing a serious concern as continuous expulsion of high phosphorus load causes pollution in the nearby water bodies (Jorquera et al., 2008). Hence bringing down the phytate in maize could be an important strategy for Zn biofortification.

Low phytic acid (*lpa*) mutants are available in maize (Raboy et al., 2000). These mutants produce seeds that have normal levels of total phosphorus but greatly reduced levels of phytic acid phosphorus. The *lpa* mutations do not affect the ability of a plant to uptake phosphorus and its transportation to a developing seed; instead, they block the ability of a seed to synthesize phosphorus into phytic acid (Pilu et al., 2003). Recessive *lpa1-1* mutation causes up to 55–65% reduction of phytic acid in maize grain and is due to a mutation in trans-membrane transporter protein (MRP). The *lpa2-1* mutation causes 50% reduction in phytic acid, and is due to a mutation in inositol phosphate kinase (IPK) enzyme (Raboy et al., 2000). *lpa241* mutation which reduces phytic acid up to 90% originated from mutation in myo-inositol(3)P1 synthase (MIPS) enzyme (Pilu et al., 2005).

Ertl et al. (1998) developed low phytate maize genotypes *via* backcross method. Beavers et al. (2015) generated low phytic acid maize population without negatively affecting seed quality through three rounds of selections in broad-based synthetic populations. Though *lpa241* showed 30% reduction in germination (Pilu et al., 2005), no negative effects on germination, initial establishment, growth, and response to pests and diseases were observed for *lpa2-1* and *lpa1*-*1* (Raboy et al., 2000). Considering this, *lpa2-1* and *lpa1*-*1* have been used in various breeding programs. In India, *lpa2-2* allele was successfully introgressed into well-adapted and productive elite inbred lines *viz.*, UMI-395 and UMI-285 through MAS (Sureshkumar et al., 2014; Tamilkumar et al., 2014). Recessive *lpa1-1* and *lpa2-1* mutants have also been combined with high provitamin-A and QPM traits in elite genetic backgrounds (Bhatt et al., 2018). Though there is no report of release of low phytate maize cultivars, the *lpa* genotypes developed by different research groups hold promise for their deployment to alleviate Zn deficiency. It is important to integrate low phytate program with breeding for high-Zn content, as the benefits of *lpa* mutant can be best realized in genotypes with higher kernel Zn. In addition to genetic interventions, phytate content can also be reduced through maize processing methods like lime-cooking and fermentation (Ekpa et al., 2018).

## Vitamin E-Enriched Maize

### Extent of Vitamin E Deficiency

Vitamin-E is an essential micronutrient in human body, and plays vital role in scavenging of various reactive oxygen species (ROS) and free radicals, quenching of singlet oxygen (high energy oxygen), and providing membrane stability by protecting polyunsaturated fatty acids (PUFA) from lipid peroxidation (Fryer, 1992). Vitamin-E helps in preventing Alzheimer’s disease, neurological disorders, cancer, cataracts, age-related macular degeneration, and inflammatory disease (Bramley et al., 2000). Supplementation of vitamin-E in the feed also ensures enhanced quality and prolongs stability of animal meat (De Winne and Dirinck, 1996; Sanders et al., 1997).

Recommended dietary allowance (RDA) for vitamin-E is 4 mg day^−1^ for 0–6 month’s old child, while the same for ≥14 years old is 15 mg day^−1^ for both males and females (Institute of Medicine, 2000). It is estimated that over 20% of the examined people both in developed and developing countries possess plasma α-tocopherol lower than the recommended level (Li et al., 2012). In a study in South Korea, two-third of adults were reported to have suboptimal vitamin-E level and almost one fourth are deficient (Kim and Cho, 2015). About one-third of the pregnant women in rural Nepal are severely affected by vitamin E deficiency (VED) (Jiang et al., 2005). In Bangladesh, VED is more critical, as about two-third of women in early pregnancy were found to be severely vitamin-E deficient (Shamim et al., 2015).

### Molecular Breeding for Vitamin E Enrichment

Wide genetic variation in tocopherol components has been observed in maize (Rocheford et al., 2002; Egesel et al., 2003; Li et al., 2012; Feng et al., 2015; Muzhingi et al., 2017; Das et al., 2019a). Estimation of tocopherol fractions (α-, β-, γ-, and δ-) is simple and fast (~15 min/sample) using HPLC, but it involves high cost (US$25–30 per sample). Selection of key genes that enhance tocopherol in maize, therefore, provides cost-effective (US$0.5/sample of PCR) solution. Several earlier studies (Wong et al., 2003; Shutu et al., 2012; Feng et al., 2013; Lipka et al., 2013; Diepenbrock et al., 2017) reported QTLs for higher accumulation of kernel α-tocopherol, γ-tocopherol, and total tocopherol in maize. The pathway for vitamin-E biosynthesis is also well-characterized (DellaPenna and Pogson, 2006). Several genes *viz.*, homogentisate phytyltransferase (*VTE2*), homogentisate geranylgeranyl transferase (HGGT), methyl transferase (*VTE3*), tocopherol cyclase (*VTE1*), phytol kinase (*VTE5*), and γ-tocopherol methyl transferase (*VTE4*), play important role in regulating the pathway. Among these genes, *VTE4* was identified as the key gene that enhances the accumulation of α‐tocopherol by converting γ-tocopherol (Li et al., 2012). Two insertion/deletions (InDel7 and InDel118) within the gene *VTE4* were found to significantly affect the level of α-tocopherol. InDel118, located 9-bp upstream of the putative transcription start site, controls α-tocopherol content by regulating *VTE4* transcript level, whereas InDel7 affects translation efficiency. Association of *VTE4* with higher accumulation of α-tocopherol was also reported by Lipka et al. (2013). Das et al. (2019b) later identified one SNP (G to A), and three InDels (14 and 27 bp) in the *VTE4* gene comprising a favorable haplotype (0/0) which can differentiate low and high α-tocopherol accumulating maize lines. These newly identified SNP and InDels in addition to the previously reported InDel118 and InDel7 can be useful in selection of favorable genotypes with higher α-tocopherol in maize.

**Figure 3:**
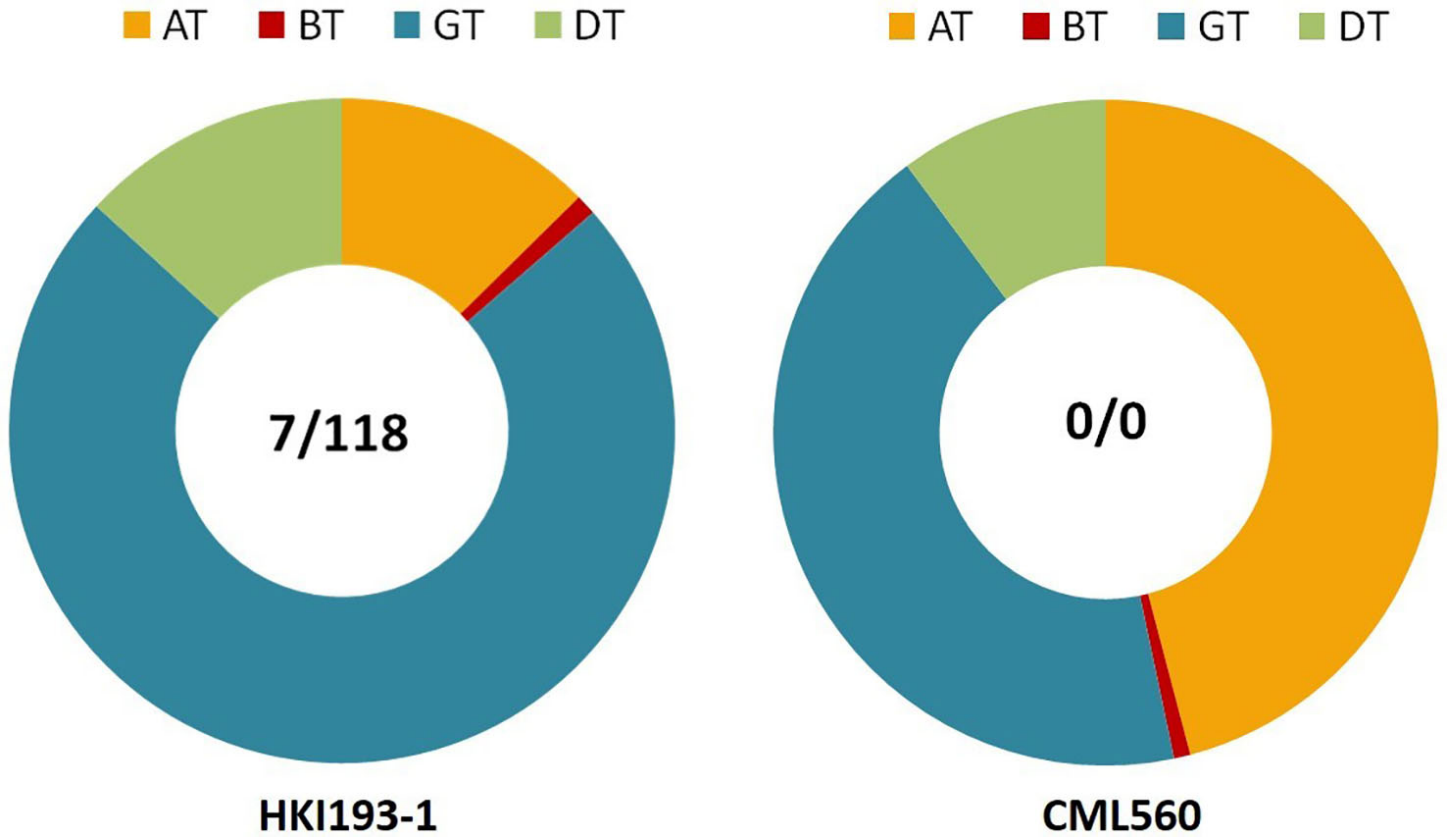
HKI193-1, an elite maize inbred with an unfavorable haplotype (7/118) for *VTE4*, showing low AT (12.6%) *versus* CML560 with a favorable haplotype (0/0) showing high AT (45.9%). AT, α-tocopherol, BT, β-tocopherol, GT, γ-tocopherol, DT, δ-tocopherol.

Das et al. (2019c) screened large number of maize inbreds of diverse pedigree using gene-based markers specific to InDel118 and InDel7 of *VTE4* and identified inbreds with favorable haplotype (0/0: deletion at InDel118 and InDel7) of *VTE4*. CML560 and CML496 possess the most favorable haplotype (0/0) for *VTE4* (**Figure 3**). Das et al. (2019b) developed hybrids using inbreds possessing the favorable haplotype of *VTE4*, and reported higher mean α-tocopherol (mean: 21.37 ppm) than the check hybrids (mean: 11.16 ppm). In some of the hybrids *viz.*, MHVTE-2, MHVTE-18, MHVTE-28, MHVTE-10, and MHVTE-3, α-tocopherol constituted ≥50% of the total tocopherol.

Considering the major effect of *VTE4* in accumulating higher α-tocopherol, Feng et al. (2015) transferred the favorable allele from a suitable donor parent (SY999) to four Chinese *shrunken2*-based sweet corn lines (M01, M14, K140, and K185) through MABB. Average increment of 7.73 ppm of α-tocopherol was observed among the MABB-derived progenies, with α-tocopherol as high as 15.99 ppm, compared to 3.14 ppm in recurrent parents. In India, favorable allele of *VTE4* has been introgressed into four provitamin-A rich QPM elite inbreds using MABB that led to the increase of α-tocopherol to 15.2 ppm over 8.0 ppm in the original inbreds (Hossain et al., 2018b). Though α-tocopherol (>30 ppm) has been reported in maize germplasm, the extent of increase due to introgression of favorable allele of *VTE4* depends on the base level expression of the other important genes in the pathway, and further interaction with background genome (Das et al., 2019c).

## Maize Nutritional Quality Analysis

High-throughput analytical methodologies are critical for integration of biofortification strategies into mainstream plant breeding as well as for assurance of the nutritional quality in the seed that reaches farmers and consumers (Guild et al., 2017). Given the fact that several of the nutritional traits are not visible to a naked eye, it is extremely important that these are measured in an appropriate laboratory where such methodologies are well-established. To achieve the level of precision required while optimizing costs, different analytical methods may need to be employed during the process of breeding for the target traits, as well as quality assessment/quality control (QA/QC) of seed of parental lines (Gowda et al., 2017) post-cultivar release (**Table 1**).

**Table 1:** Analytical methods used at different breeding stages for the targeted maize biofortification traits at International Maize and Wheat Improvement Center (CIMMYT).

Trait	Germplasm screening/breeding stage^a^	Analytical methods^b^	Cost per sample (USD)
Provitamin A	Landraces; new germplasm; stages 1–2; QA/QC of seed post-cultivar release	NIRS (for total carotenoids)	2.8
Kernel zinc	Stage 3; variety release and promotion Landraces; new germplasm; stages 1–2;	HPLC/UPLC XRF	38.3 5.8
QPM	QA/QC of seed post-cultivar release		
	Stage 3; variety release and promotion	ICP-OES	13.7
	Landraces; new germplasm; stages 1–2;QA/QC of seed post-cultivar release	NIRS	2.8
	Stage 3; cultivar release and promotion	Colorimetric methods	18.9

a QA/QC, quality assessment/quality control.

b NIRS, near-infrared spectroscopy; HPLC, high performance liquid chromatography; UPLC, ultra performance liquid chromatography; XRF, X-ray fluorescence; ICP-OES, inductively coupled plasma–optical emission spectroscopy.

Near infrared spectroscopy and X-ray fluorescence methodologies can be effectively used for early breeding generations to identify and reject materials that have low values of PVA. However, for advanced generation materials or commercial samples, it is always recommended to conduct the wet chemistry analysis, using chromatography and inductively coupled plasma optical emission spectroscopy (ICP-OES).

Garcia-Oliveira et al. (2018) pointed out that apart from the actual germplasm and environmental influence on kernel-Zn concentration, there could be numerous possibilities for introducing variation in the results of different studies, ranging from sensitivity of the method used for the quantification of Zn contents, improper postharvest handling of the samples, and the significant variability among microenvironments for Zn. Spectroscopic methods, such as ICP-OES and atomic absorption spectroscopy (AAS), are therefore used to provide robust and accurate results on kernel Zn content in maize. Limits of detection span a wide analytical range from percentage by weight to parts per billion (ppb) levels. Sample digestion can be done chemically or by using micro-wave methods, which increase the processing throughput prior to analysis. For early stages of high-Zn maize breeding, where large number of samples are screened, X-ray fluorescence (XRF) is extensively used (Hindu et al., 2018; Palacios-Rojas et al., 2018). The cost of analysis per sample by XRF is typically lower [approx. 6 US dollars (USD)] than ICP-OES (approx. 14 USD) (**Table 1**). Additional benefits of the method include simplicity, avoiding the use of hazardous chemicals, and less costly sample preparation.

Unlike organic micronutrients, such as PVA carotenoids, degradation is not an issue for high-Zn maize grains; however, the potential for contamination during the sample manipulation and analysis is high due to the high abundance of Zn in the environment. In the case of maize, use of flour is required due to the heterogeneity in grain shape; use of flour also improves data reproducibility and accuracy compared with whole grain analysis. It is important to ensure that grinding is performed using a grinder with non-contaminating material such as zirconium. When ICP-OES is performed, aluminum determination is used as an indicator of sample contamination (Guild et al., 2017).

## Nutritionally Enriched Maize Cultivar Releases and Commercialization

**Figure 4:**
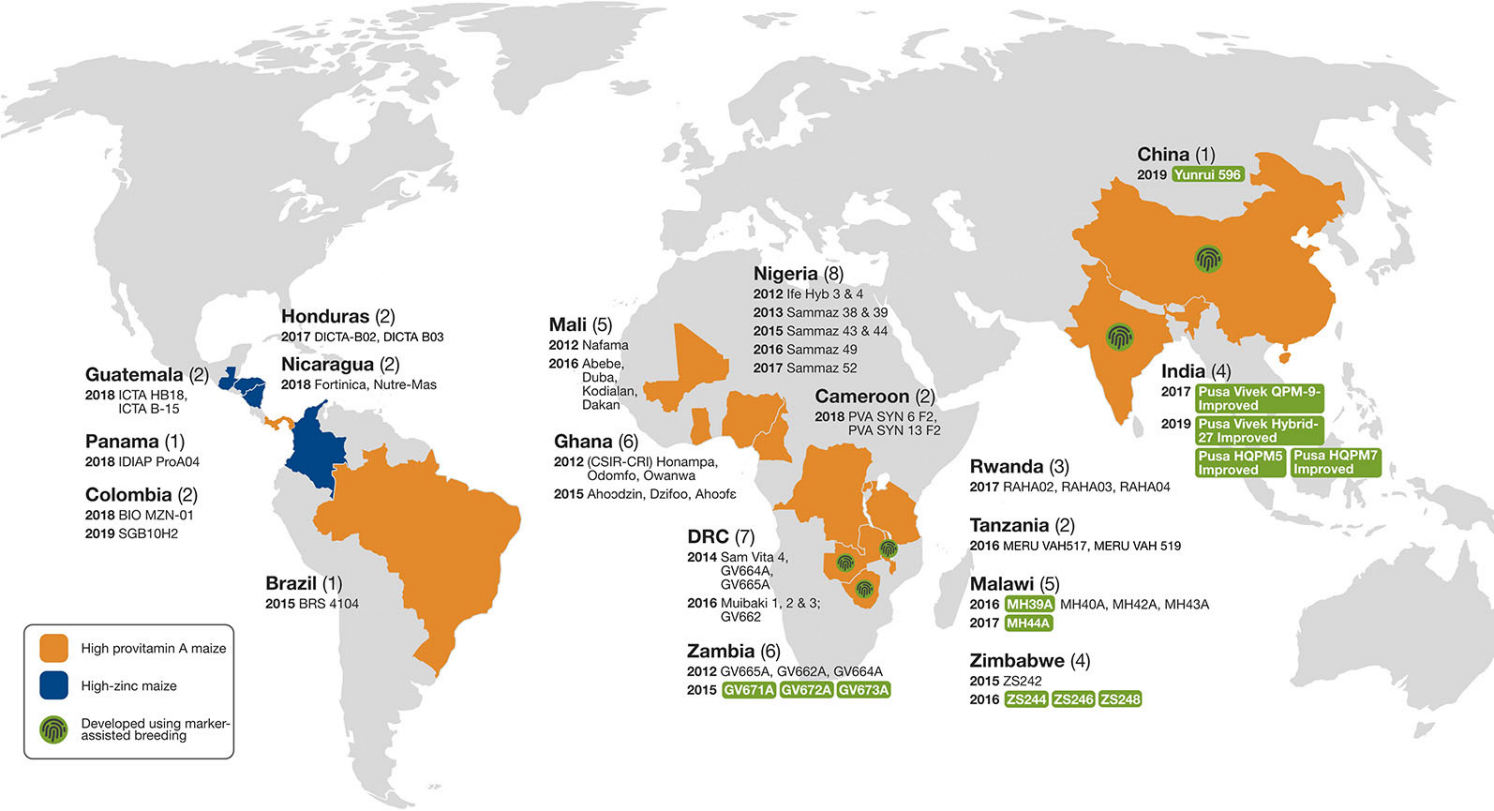
Provitamin A-enriched and high-Zn maize cultivars developed using conventional and molecular marker-assisted breeding and released for commercial cultivation in sub-Saharan Africa, Asia, and Latin America (modified from Listman et al., 2019).

Tropical maize inbred lines with enhanced nutritional quality (especially QPM, PVA, and high-Zn) and other desirable agronomic and adaptive traits developed at CIMMYT and IITA have been used in several countries to develop agronomically competitive hybrids or OPVs or synthetics (Menkir et al., 2018). **Figure 4** shows the varietal release status with regard to provitamin A and high-Zn maize in SSA, Asia, and Latin America.

Extensive multi-location evaluations of PVA-enriched OPVs and hybrids in collaboration with public and private sector partners in Zambia, Zimbabwe, Tanzania, Ghana, Nigeria, Mali, Malawi, and the Democratic Republic of Congo (DRC) led to the selection of promising hybrids and synthetics for further evaluation in national performance trials (NPTs) and farmer participatory on-farm trials. These testing schemes led to the release of 47 PVA-enriched hybrids and synthetics that meet 50–80% of the current PVA breeding target in nine countries, including Zambia, Zimbabwe, Malawi, Tanzania, DRC, Ghana, Mali, and Nigeria (Menkir et al., 2018). In addition, the high PVA inbred lines developed at CIMMYT and IITA have been effectively used as PVA donors by the national maize breeding programs in Asia (e.g., China and India) and Latin America (e.g., Brazil and Panama) to develop improved, nutritionally enriched maize cultivars (Muthusamy et al., 2014; Liu et al., 2015; Zunjare et al., 2018; Goswami et al., 2019; Listman et al., 2019).

The first PVA-enriched maize hybrids and synthetics released in SSA in 2012 had an average PVA content of about 6.0 to 7.5 µg g^−1^, or 40–50% of the breeding target. Since then several hybrids exceeding 10 µg g^−1^ PVA have been released (Andersson et al., 2017). A hybrid with >90% of the PVA breeding target (14.1 µg g^−1^) was released in Malawi in 2016. In each of the target countries, PVA-enhanced cultivars are released either by the national research institutes or by private seed companies working in those countries.

HarvestPlus and national governments in SSA have invested significant effort in creating awareness and consumer demand in the target countries where market preference is for white maize. These efforts seem to be paying off; Zambia now has more than 500 tons of certified seed of PVA maize cultivars, covering an estimated 200,000 ha. Further, linkages were established with millers and food processing companies to ensure that farmers have access to markets for their excess grain. Despite the awareness campaigns, school feeding schemes that are funded by non-government organizations (NGOs) and the government remain a substantial part of the market for high PVA maize, raising questions about what will happen when that support is not sustained. Influencing national policy on balanced nutrition and health appears to be the most viable way to create and sustain demand for PVA-enhanced maize because most governments in the target countries provide subsidies to farmers and buy most of the grain as part of a national food security strategy (Simpungwe et al., 2017).

CIMMYT, in collaboration with public and private sector partners in Mexico, Guatemala, Nicaragua, El Salvador, Honduras, and Colombia, has been working on development and deployment of elite high-Zn maize cultivars in Latin America. Extensive multi-location trials showed relative yield parity and similar performance for other agronomic traits, relative to commercial checks, indicating the competitiveness of the these products in the lowland tropics of Latin America. So far, four high-Zn maize cultivars (two hybrids and two synthetics) have been released in Latin America. These cultivars have 90 to 110% of the target kernel Zn content set under HarvestPlus and are competitive for grain yield and other adaptive traits with the commercial checks (Listman et al., 2019).

**Figure 5:**
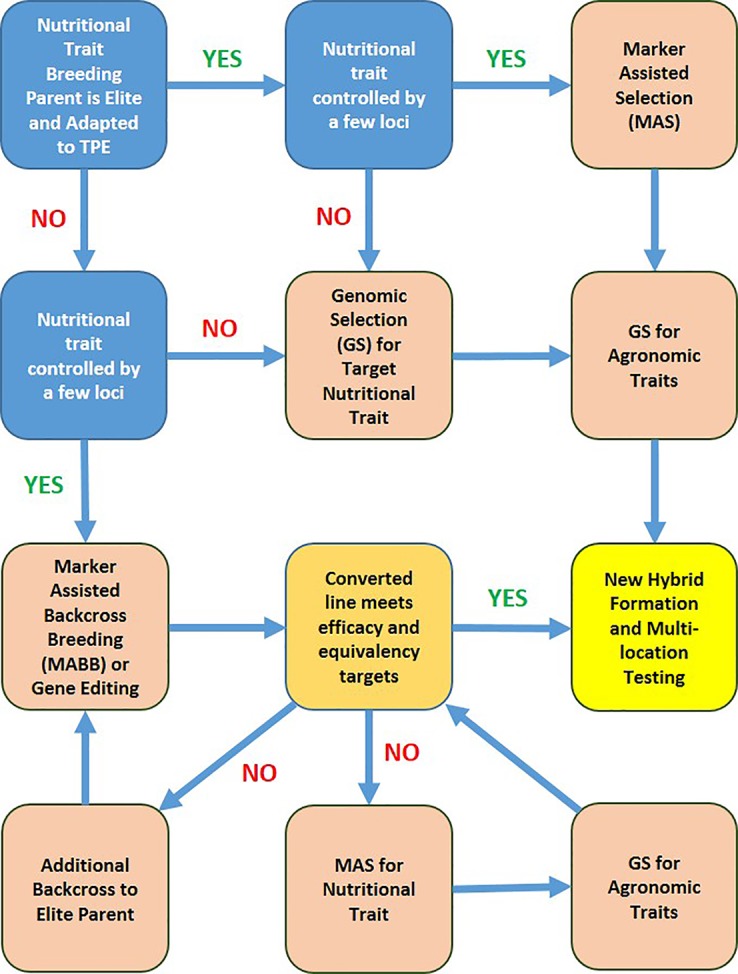
A schematic depicting the strategy with decision tree for molecular marker-assisted breeding workflows to accelerate progress toward nutritional trait targets. TPE refers to the target population of environments of the product profile with the target nutritional trait(s).

A schematic depiction of decision tree for molecular marker-assisted breeding workflows to accelerate progress toward nutritional trait targets is shown in **Figure 5**. The workflow decisions are based on relative eliteness and adaptation of initial nutritional trait donor breeding parent, genetic complexity of the target nutritional trait, and relative cost of phenotypic assays, trait-linked marker assays, and genome profiling. Efficacy of a converted line *via* MABB or gene editing refers to the nutrient level of the converted line relative to the target. Equivalency of a converted line refers to performance of the new version relative to the elite recurrent parent for important agronomic and adaptive traits.

In recent years, elite maize inbred lines and hybrids with multiple nutritional quality traits, especially combinations of QPM, provitamin A, low phytate, and vitamin E traits, have been developed in India and China, through molecular marker-assisted breeding (**Table 2**). Stacking multiple traits essentially requires raising of large population size in MABB program compared to a population segregating for one target gene. Since traits like QPM, provitamin A, vitamin E, and kernel Zn are not associated with yield penalty, they can be generally stacked together. One of the elite hybrids with a stack of nutritional quality traits (QPM and Provitamin A), Pusa Vivek QPM-9 Improved, developed by ICAR-Indian Agricultural Research Institute has been officially released in India in 2017 for commercial cultivation. Three MABB-derived hybrids, including one provitamin A hybrid, Pusa Vivek Hybrid-27 Improved, and two QPM + provitamin A-enriched hybrids, Pusa HQPM5 Improved and Pusa HQPM7 Improved, have been approved for release in India in 2019. High provitamin A trait has been introgressed in both traditional and QPM genetic backgrounds (Muthusamy et al., 2014; Andersson et al., 2017). In addition, the germplasm resources developed through stacking of provitamin A and vitamin E could be potentially used for analysis of carotenoid stability.

**Table 2:** Some examples of stacking of nutritional quality traits in maize using molecular marker-assisted breeding.

Traits	Gene combination	Nutritional trait values	Improved genotypes developed	References	
QPM + provitamin A	*CrtRB1* in a QPM hybrid (Vivek QPM9)	8.16 µg g^−1^ PVA; 0.74% Trp; and 2.67% Lys (Lys and Trp as % endosperm protein)	Pusa Vivek QPM9 Improved	Muthusamy et al. (2014); Yadava et al. (2018)
	*CrtRB1* in QPM inbreds (CML161 and CML171)	5.25 to 8.14 µg g^−1^ PVA; 0.35% Lys in endosperm flour	Provitamin A-enriched elite QPM inbreds CML161 and CML171	Liu et al. (2015)
	*CrtRB1*, *LcyE*, and *o2* in QPM inbreds	9.25–12.88 µg g^−1^ PVA; 0.334% Lys and 0.080% Trp (Lys and Trp estimated in endosperm flour)	Pusa HQPM-5 Improved; Pusa HQPM-7 Improved	Zunjare et al. (2018)
	*CrtRB1* in a QPM inbred	10.75 µg g^−1^ PVA; 0.303% Lys and 0.080% Trp (Lys and Trp estimated in endosperm flour)	Provitamin A-enriched elite QPM inbred HKI1128Q (parent of Pusa HM9 Improved, HM10Q, and HM11Q)	Goswami et al. (2019)
QPM + provitamin A + vitamin E	*CrtRB1, LcyE*, and *VTE4* in QPM background	16.8 µg g^−1^ alpha-tocopherol; 11.5 µg g^−1^ PVA; 0.367% Lys and 0.085% Trp (Lys and Trp estimated in endosperm flour)	Improved versions of QPM and provitamin A rich hybrids (HQPM-1-PV, HQPM-4-PV, HQPM-5-PV, and HQPM-7-PV)	Hossain et al. (2018b)
QPM + provitamin A + low phytate	*lpa1-1* and *lpa2-1* in provitamin A-enriched QPM lines	8.3–11.5 µg g^−1^ PVA; 0.323–0.372% Lys and 0.081–0.087% Trp (Lys and Trp estimated in endosperm flour); 30–40% reduction in phytic acid P	Improved versions of elite inbreds (HKI161-PV, HKI163-PV, HKI193-1-PV, and HKI193-2-PV)	Bhatt et al. (2018)	

Genome editing represents a powerful technology for enhancing and stacking nutritional quality traits in maize. Liang et al. (2014) reported editing of the genes involved in phytic acid synthesis (*ZmIPK1A*, *ZmIPK*, and *ZmMRP4*) in maize. Since several of the important genes influencing nutritional quality traits, such as QPM and provitamin A, have been well-characterized, gene editing could provide a powerful system for stacking these traits in agronomically superior genetic backgrounds. For example, if one wants to stack a PVA trait in an elite QPM line, editing the elite line for *CrtRB1* could potentially simplify the breeding process. Similarly, it should be possible to design a PVA target and edit two to four key carotenoid biosynthetic pathway genes in elite climate-resilient maize germplasm.

## Concluding Remarks

Challenges in agriculture and food production keep evolving. In the 1950s, the world needed a large boost in food production to combat famine, in the face of rapid population growth and recurring natural disasters. In the 21st century, the challenge is not only to produce enough to feed the growing population, but also providing nutritionally balanced diets. Moving away from overemphasis on calorie security, the food today on everyone’s plates must be of appropriate quantity, nutritious, and produced in an environmentally, economically, and socially sustainable manner. The EAT-Lancet Commission Report (Willett et al., 2019) highlighted the importance of promoting diets that are nutritious and which can reduce the environmental impact of food systems. The sum of different agricultural and nutrition sensitive strategies could contribute to sustainable and nutritional food systems.

Although not a silver bullet solution, biofortification has proven to be an efficient strategy to combat malnutrition. CIMMYT, IITA, and national partners [especially in Africa, Asia, and Latin America (LatAm)] have employed conventional breeding and molecular tools, to successfully develop and release several nutritious maize cultivars without compromising grain yield levels or other important agronomic and adaptive traits. Many of these biofortified maize cultivars are currently grown by farmers and accepted by consumers in many countries (Talsma et al., 2017; Manjeru et al., 2019). Advances in phenotyping coupled with molecular breeding facilitated achievement of the breeding targets for various nutrients in maize. Going forward, the focus should be on mainstreaming breeding for nutrient enrichment into maize breeding efforts to deliver high-performing climate-resilient maize cultivars with improved nutritional quality to farmers and consumers. Efforts need to be made to develop elite maize cultivars stacked with multiple nutrients to address multiple nutrient deficiencies that are prevalent especially in SSA, LatAm, and Asia, as well as in combining biofortification with complementary strategies like dietary diversification, and enhancement of nutrients through agronomic and/or food processing interventions.

Nutrition and health have become the main factors influencing people’s diet. The combinations of nutritional quality traits, including QPM, PVA, high-Zn, etc. in both maize grain and in fresh corn has consumer appeal, and contributes to national initiatives and sustainable development goals for enhancing nutrition. The rapid advances that have been made in understanding the genetic control of many macro- and micro-nutrients in maize grains, coupled with the availability of new tools/technologies such as genomic selection, will accelerate the rate of genetic gain for improved nutrient content in maize.

Specialty maize including sweet corn, waxy corn, and popcorn has experienced tremendous growth during the last 30 years, and improving its nutritional quality will stimulate further development of this market. The key to the future development of biofortified specialty maize cultivars is to continue to increase the level of heterosis, seed production, and diversification of the products to meet the changing dietary needs and consumer preferences.

Interdisciplinary work and more effective integration of national and international research efforts are key for enhanced development and dissemination of biofortified crops. For biofortified maize cultivars to succeed in the market, it is important to understand the market dynamics. Value chains that effectively link the farmers to the processors and the consumers need to be improved. Only through such linkages can the value of biofortified crop cultivars can be fully exploited, malnutrition alleviated, and new markets opened.

## Author Contributions

BP developed the outline for the review, and synthesized the manuscript, based on the contributions from all the co-authors (NP-R, FH, VM, AM, TD, TN, FV, SN, BV, XZ, MO and XF).

## Funding

The work presented in this review article was supported by various projects, especially the HarvestPlus Program, and the CGIAR Research Program on Maize (MAIZE). MAIZE receives Windows 1&2 support from the Governments of Australia, Belgium, Canada, China, France, India, Japan, Korea, Mexico, the Netherlands, New Zealand, Norway, Sweden, Switzerland, the UK, the USA, and the World Bank. The work on maize biofortification in India has been supported by the Indian Council of Agricultural Research (ICAR); Department of Biotechnology (DBT), Government of India; SERB-Department of Science and Technology (DST), Government of India; and ICAR-All-India Coordinated Research Project (Maize).

## Conflict of Interest

The authors declare that the research was conducted in the absence of any commercial or financial relationships that could be construed as a potential conflict of interest.
